# Mast cells enhance venous thrombus stability and lung fibrin deposition in murine sepsis

**DOI:** 10.1182/bloodadvances.2026020424

**Published:** 2026-09-04

**Authors:** Julie Rayes, Yuancheng Luo, Amit S. Bhopal, Katharine Whitworth, Asif J. Iqbal, Alexander Brill

**Affiliations:** Department of Cardiovascular Sciences, School of Medical Sciences, College of Medicine and Health, University of Birmingham, Birmingham, United Kingdom

## Abstract

Venous thromboembolism is a serious complication of sepsis despite standard thromboprophylaxis. Mast cells (MCs) promote deep vein thrombosis (DVT) in sterile murine models, but their contribution during sepsis remains unknown. We explored the role of MCs in DVT in lipopolysaccharide (LPS)-induced sepsis in mice. We show that LPS treatment does not alter DVT prevalence or size in control C57BL/6 mice but increases intrathrombus inflammatory mediators, including interleukin-1β (IL-1β) and citrullinated histone 3 (CitH3), a hallmark of neutrophil extracellular traps (NETs). MC ablation (KIT^W-sh^ mice) protected animals from sterile DVT for up to 1 week, whereas in endotoxemia settings, KIT^W-sh^ mice produced thrombi similar to controls. Thrombi from KIT^W-sh^ mice were more susceptible to lysis by a combination of tissue plasminogen activator and DNase I and contained less CitH3 than controls. Septic DVT exacerbated pulmonary thromboinflammation characterized by large fibrin deposits and high levels of CitH3 and IL-1β in control but not KIT^W-sh^ mice. MC deficiency during septic DVT was associated with decreased plasma angiopoietin-2 levels, suggesting improved endothelial stability and reduced vascular inflammation. Thus, in septic conditions, MCs are dispensable for thrombus initiation but promote NET-rich thrombus stability, resistance to lysis, and pulmonary thromboinflammation, the major life-threatening complication of DVT.

## Key Points

• MCs are dispensable for septic DVT initiation but regulate thrombus composition and stability.
• MCs promote NET-rich thrombi resistant to lysis and drive pulmonary thromboinflammation in septic DVT.

## Visual Abstract

**Figure:**
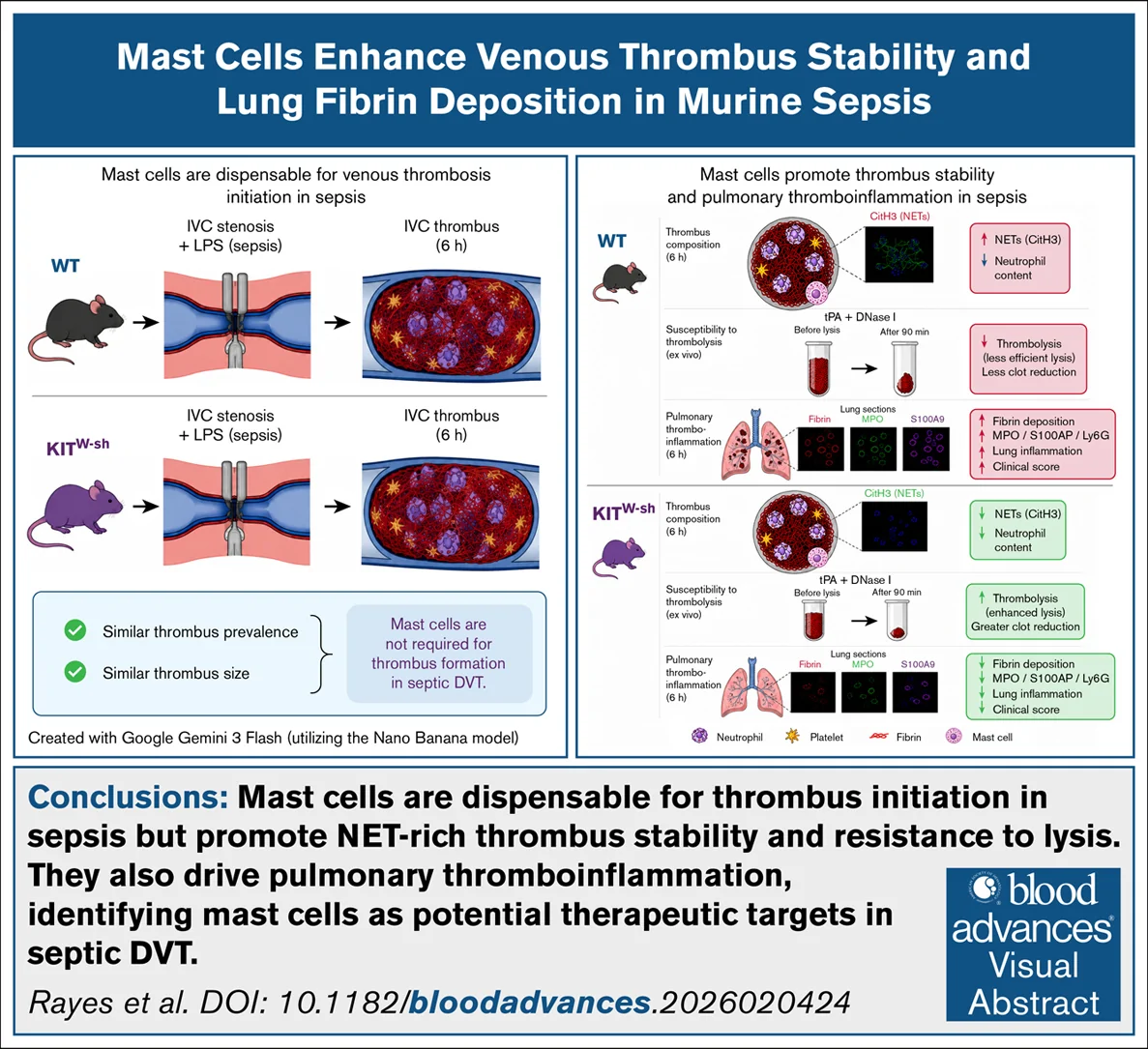

## Introduction

Venous thromboembolism (VTE), including deep vein thrombosis (DVT) and the most serious complication, pulmonary embolism, occurs at an annual incidence of about 1 per 1000 adults, and VTE is the third leading cause of vascular death after myocardial infarction and stroke.^1^ DVT is characterized by the formation of blood clots in deep veins, most commonly in the lower limbs, and these clots can dislodge and obstruct the pulmonary circulation, leading to pulmonary embolism, which may cause severe respiratory failure and is the first manifestation of disease in about 25% of patients.^2^ The pathogenesis of DVT is multifactorial and strongly associated with reduced blood flow due to immobilization, surgery, or prolonged travel.^3^ Experimental studies have shown that venous thrombosis triggered by blood flow stagnation involves inflammation-like processes, including endothelial activation and recruitment of leukocytes and platelets.^4^^,^^5^ Recent work has identified mast cells (MCs) as important contributors to venous thrombosis. Our group demonstrated that MCs promote DVT formation in mice,^6^ whereas other studies showed that MC-derived chymase plays a key role in this process.^7^

Venous thrombi are inflammatory structures, with their formation, propagation, and stability intimately linked to cellular and soluble inflammatory mediators. Neutrophil extracellular traps (NETs), networks of DNA, histones, and damage-associated molecular patterns released by activated neutrophils, are a critical component of venous thrombi, providing structural support to fibrin and promoting thrombosis.^8^, ^9^, ^10^ NET formation is closely linked to activation of the NLRP3 (NOD-, LRR- and pyrin domain-containing protein 3) inflammasome and release of proinflammatory cytokines such as interleukin-1β (IL-1β), creating a positive feedback loop that enhances thromboinflammation.^11^

VTE is not restricted to sterile environments but is a major complication associated with sepsis. Venous thromboembolism occurs in ∼40% of critically ill patients with sepsis despite standard thromboprophylaxis, yet the cellular mechanisms driving sepsis-associated thrombosis remain incompletely understood.^12^ Therefore, we investigated how MCs influence DVT formation, thrombus composition, and associated pulmonary thromboinflammation in a murine model of endotoxemia induced by lipopolysaccharide (LPS).

## Methods

KIT^W-sh^ MC-deficient mice were from The Jackson Laboratory on a C57BL/6 background. To model DVT, stenosis of the inferior vena cava (IVC) over a 30G needle spacer was applied; all visible side branches were closed, and then the spacer was removed.^13^ All animal experiments were performed in accordance with the Animals Scientific Procedures Act 1986, conform to the guidelines from Directive 2010/63/EU of the European Parliament on the protection of animals used for scientific purposes, and were approved by the local ethical committee and the UK Home Office (project license PP8612246). For ex vivo thrombolysis, thrombi were placed in the solution of tissue plasminogen activator (tPA) or DNase I, or both in 1 mL saline and incubated on a shaker; the weight of thrombi was measured every 30 minutes. Plasma cytokine levels were measured by Luminex technology. Detailed methods are included in the supplemental Methods.

## Results

### MCs are dispensable for initiation of septic DVT

We used the IVC stenosis model because residual venous blood flow involves thromboinflammatory mechanisms central to this study. Although LPS has been reported to enhance thrombosis in complete stasis models, these models rely on fundamentally different mechanisms of thrombogenesis and are therefore not directly comparable.

We first assessed whether MCs influence the initiation of DVT during endotoxemia. Under sterile conditions, MC-deficient KIT^W-sh^ mice were strongly protected from thrombosis for up to 8 days after IVC stenosis (Figure 1A), consistent with a previous study demonstrating a critical role of MCs in thrombus initiation.^6^ In contrast, during LPS-induced sepsis, MC deficiency did not alter thrombosis prevalence or thrombus size compared with wild-type (WT) controls (Figure 1B-D). These findings indicate that, although MCs are essential drivers of sterile thrombogenesis, inflammatory cues during sepsis introduce alternative prothrombotic pathways that override MC-dependent initiation mechanisms. Blood platelet counts were significantly higher in KIT^W-sh^ mice than in controls both with and without LPS, and systemic LPS administration reduced circulating leukocyte counts and altered platelet numbers similarly in both genotypes (supplemental Figure 1), supporting the concept that the global inflammatory response remained intact despite MC ablation. The LPS protocol used in this study was designed to model acute endotoxemia-associated thromboinflammation rather than the full spectrum of human polymicrobial sepsis and produced hematological changes characteristic of systemic inflammatory activation, including leukopenia and thrombocytopenia (supplemental Figure 1A-E). Collectively, these observations suggest that MCs are dispensable for the onset of septic DVT but may influence later stages of thrombus development.

**Figure 1.**
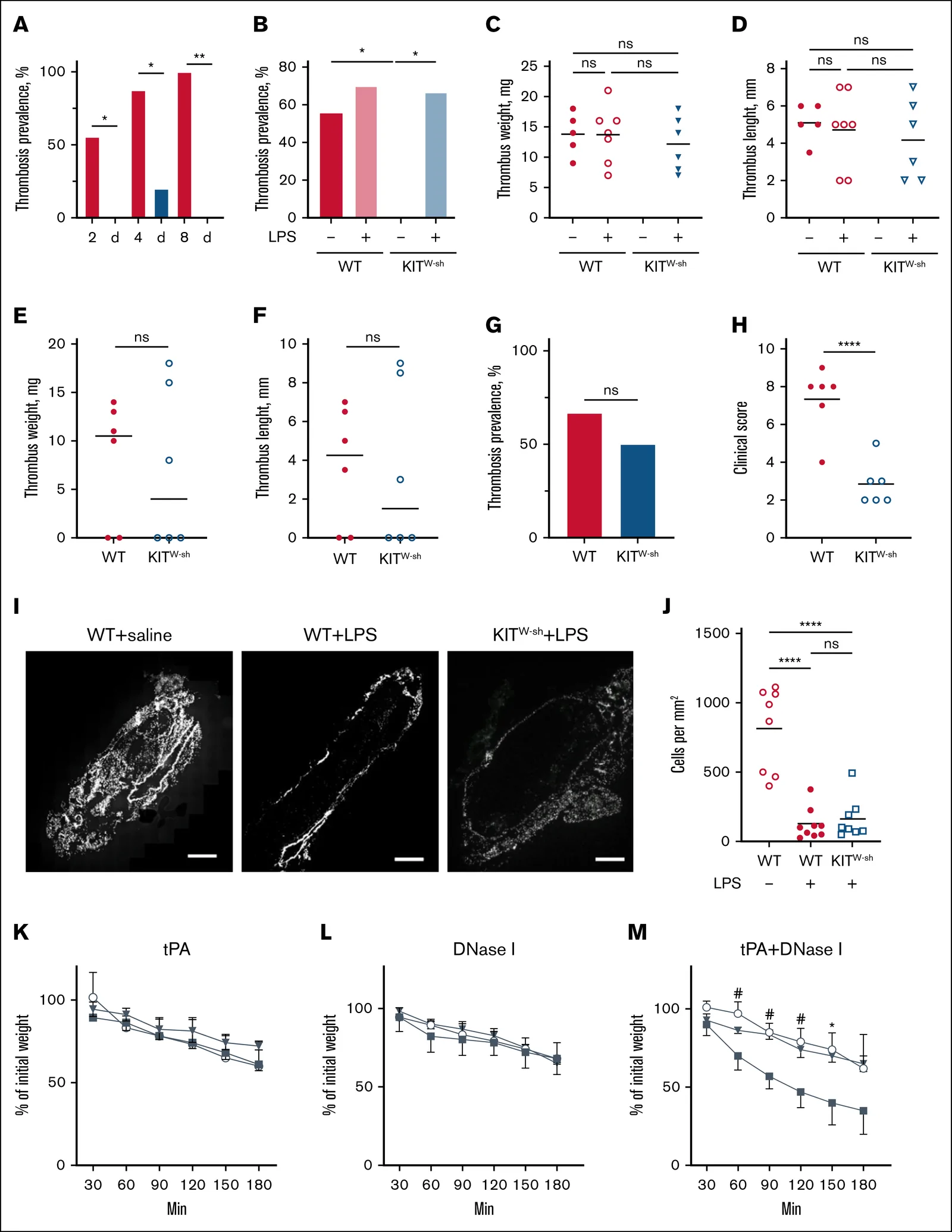
**MCs are dispensable for septic DVT initiation but promote thrombus resistance to lysis.** (A) Thrombosis prevalence in unchallenged WT (red columns) or KIT^W-sh^ (blue columns) mice subjected to IVC stenosis for 2, 4, or 8 days. MC deficiency strongly reduced DVT formation under sterile conditions; n = 8 to 10 mice per group. (B-D) WT or KIT^W-sh^ mice were administered intraperitoneal saline or LPS (4 mg/kg) and subjected to 6 hours of IVC stenosis. (B) Thrombosis prevalence, (C) thrombus weight, and (D) thrombus length are presented. Thrombus weight and length were quantified only in mice that developed a thrombus; n = 5 to 7 mice per group. Statistics were performed using contingency tables followed by Fisher exact test (A-B) and 1-way analysis of variance (ANOVA) with Tukey multiple-comparisons test (C-D); ∗*P* < .05; ∗∗*P* < .01. (E-H) WT vs KIT^W-sh^ mice administered with LPS and underwent DVT surgery for 24 hours; (E) thrombus weight, (F) thrombus length, (G) thrombosis prevalence, (H) clinical score; (G) exact Fisher’s test and (E-F,H) Student unpaired *t* test were used for statistical comparison; n = 6 per group. Representative composite images of thrombi stained for nuclei (I) and quantitation of nucleated cells within thrombi (J) from WT and KIT^W-sh^ mice administered with saline or LPS before 6 hours of IVC stenosis. Bar, 500 μm; 1-way ANOVA, ∗∗∗∗*P* < .001; n = 8-9 (K-M) WT mice, pretreated (black triangles) or not (open circles) with cetirizine, and KIT^W-sh^ mice (black squares) received LPS and then underwent IVC stenosis. In 6 hours, thrombi were excised and incubated with tPA (3 μg/mL; K), DNase I (40 U/mL; L), or their combination (M) at 30°C for 3 hours. Thrombi were weighed every 30 minutes and expressed as a percentage of initial weight. One-way ANOVA with Tukey multiple-comparisons test for each time point: #*P* < .05; ∗*P* < .01; n = 3 to 4 per group. ns, nonsignificant.

To determine whether the lack of effect of MC deficiency on septic thrombosis persisted beyond the early phase, we extended the observation period to 24 hours after IVC stenosis in LPS-treated mice. Thrombus size and prevalence remained comparable between WT and KIT^W-sh^ mice at this later time point (Figure 1E-G), supporting the conclusion that MCs are dispensable for thrombus formation during septic DVT even beyond the initial stages. Notably, however, WT mice displayed substantially more severe clinical manifestations of sepsis than KIT^W-sh^ mice, with significantly higher clinical scores (7.3 ± 1.6 vs 2.8 ± 1.07; *P* < .001; Figure 1H), suggesting that MCs contribute to systemic inflammatory severity despite having limited effects on thrombus burden. Although these data do not directly assess long-term thrombus resolution, they argue against rapid spontaneous disappearance of thrombi in KIT^W-sh^ mice during the early phase after thrombosis.

### MCs regulate thrombus composition and resistance to lysis

Although thrombus prevalence was unchanged, the composition and biological behavior of thrombi differed markedly depending on MC presence. LPS exposure reduced nucleated cell numbers within thrombi (Figure 1I-J), possibly reflecting redistribution of leukocytes to peripheral organs such as the lungs, a phenomenon consistent with systemic inflammatory activation. Additional immunostaining identified Ly6G-positive neutrophils as a major cellular population contributing to this reduction (supplemental Figure 1F-I). Importantly, thrombi formed in MC-deficient mice were substantially more susceptible to ex vivo thrombolysis when treated with a combination of tPA and DNase I, whereas lysis with either agent alone was comparable between genotypes (Figure 1K-M). Because catheter-directed thrombolysis remains a major therapeutic strategy in DVT,^14^ these results highlight a functional role of MCs in shaping thrombus resistance to enzymatic breakdown rather than controlling thrombus formation itself. Interestingly, treatment of WT mice with the H1 receptor antagonist cetirizine did not increase thrombus susceptibility to ex vivo tPA/DNase-mediated thrombolysis (Figure 1K-M), suggesting that histamine signaling alone does not account for the altered thrombus stability associated with MC deficiency.

Because tPA-mediated fibrinolysis depends on plasminogen, we additionally assessed plasminogen incorporation into thrombi. No differences were detected between WT and KIT^W-sh^ mice in plasminogen-positive area or the proportion of plasminogen colocalized with fibrin (supplemental Figure 2A,C-D), indicating that the enhanced susceptibility of KIT^W-sh^ thrombi to lysis is unlikely to result from altered clot-associated plasminogen. Together, these findings indicate that the enhanced susceptibility of KIT^W-sh^ thrombi to tPA/DNase-mediated lysis reflects intrinsic differences in thrombus composition and stability rather than differences in clot-associated plasminogen.

### MCs are associated with increased NET accumulation in septic thrombi

Mechanistically, our data (in particular, the efficacy of DNase I in the lysis mix) support the concept that MCs modulate NET formation within the thrombus. NETs, characterized by citrullinated histone 3 (CitH3), are known structural components that stabilize venous clots and enhance fibrin architecture.^8^, ^9^, ^10^ LPS treatment did not change intrathrombus fibrin content but increased CitH3 levels in WT mice but not in KIT^W-sh^ animals (Figure 2), despite similar overall leukocyte presence. This finding is consistent with the possibility that MCs contribute to neutrophil activation or NET formation during septic thrombosis. Other neutrophil/NET markers, S100A9 and MPO, showed a trend toward decrease after LPS treatment in WT mice and were reduced in KIT^W-sh^ animals (supplemental Figure 2B,E-F), likely reflecting the overall reduction in thrombus leukocyte content. MC-derived mediators such as histamine, tumor necrosis factor-α, or IL-1β can promote NET formation,^15^^,^^16^ and MC extracellular traps themselves have been identified in thrombotic lesions,^17^^,^^18^ providing plausible mechanisms by which MCs alter thrombus composition. In this context, MCs may act as local amplifiers of neutrophil activation within the thrombus microenvironment, promoting the formation of a DNA-rich scaffold that reinforces fibrin networks. Consequently, MCs appear to regulate thrombus “quality,” including NET content and susceptibility to fibrinolysis. The selective enhancement of thrombolysis in KIT^W-sh^ thrombi by combined tPA and DNase I suggests that thrombus stability depends on cooperative interactions between fibrin and NETs. Because fibrin content and clot-associated plasminogen incorporation were comparable between genotypes, the increased susceptibility of KIT^W-sh^ thrombi to lysis is unlikely to reflect altered fibrinolytic substrate availability. Instead, reduced NET content may weaken the fibrin-NET scaffold, rendering thrombi susceptible to efficient degradation only when both fibrin and extracellular DNA are targeted simultaneously. Although this mechanism requires direct validation, it provides a plausible explanation for the selective efficacy of combined tPA and DNase I treatment.

**Figure 2.**
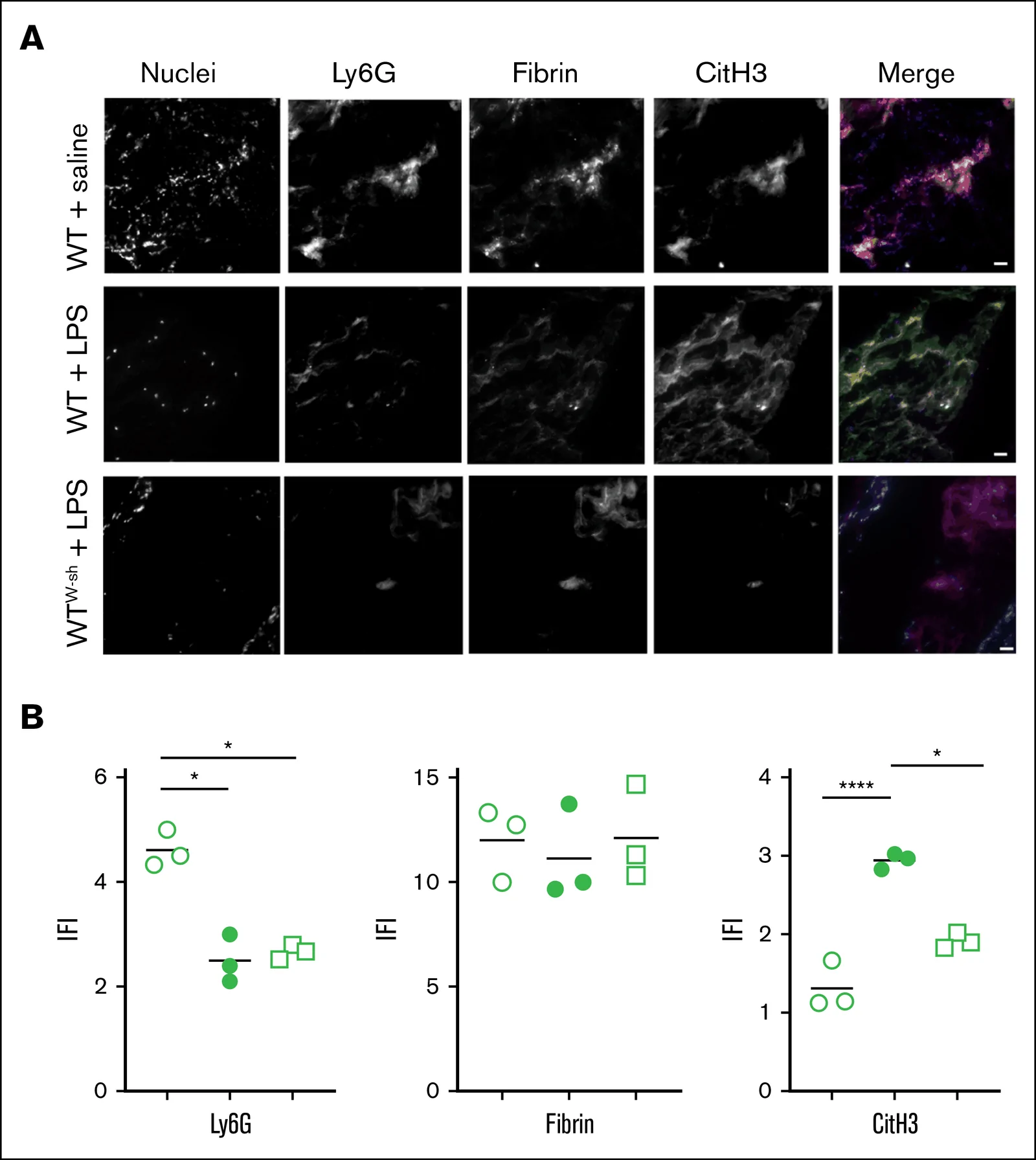
**Thrombi from KIT^W-sh^ mice contain lower levels of NETs than WT controls.** (A) Thrombi from WT mice administered with saline or LPS (4 mg/kg) and KIT^W-sh^ mice administered with LPS and then subjected to IVC stenosis were immunostained for neutrophils (Ly6G), fibrin, and CitH3. (B) Integrated fluorescence intensity (IFI) quantitation performed by ImageJ. Open circles indicate WT + saline; filled circles, WT + LPS; open squares, KIT^W-sh^ + LPS; and bar represents the mean. In the merge panel, blue indicates nuclei, red indicates Ly6G/neutrophils, magenta indicates fibrin, and green indicates CitH3; bar, 50 μm. (B) Statistical analysis was performed using 1-way ANOVA with Tukey multiple-comparisons test; ∗*P* < .05, ∗∗∗∗*P* < .004; n = 3.

### MCs promote pulmonary thromboinflammation during septic DVT

Beyond local thrombus effects, septic DVT induced pronounced pulmonary thromboinflammation. LPS administration combined with IVC stenosis increased fibrin deposition, inflammatory markers, and CitH3 in the lungs (Figure 3) of WT mice, consistent with previous studies demonstrating thromboinflammatory responses in the pulmonary circulation during thrombosis.^19^ Importantly, the increase in fibrin deposition was not observed in mice treated with LPS alone (Figure 3A), indicating that pulmonary thromboinflammation required the combination of systemic endotoxemia and venous thrombosis. These changes were markedly attenuated in MC-deficient mice, indicating that MCs facilitate distal organ inflammation during septic thrombosis. Increased pulmonary CitH3 and IL-1β levels in WT animals suggest activation of inflammasome-NET feedback loops previously described in venous thrombosis.^11^ The normalization of these markers in KIT^W-sh^ mice implies that MCs amplify systemic inflammatory signaling that extends beyond the primary thrombus site. Similarly to CitH3, pulmonary S100A9 and myeloperoxidase levels also increased after LPS challenge in WT but not in KIT^W-sh^ mice (supplemental Figure 3A,E-F). Interestingly, LPS treatment reduced pulmonary plasminogen content, and the proportion of plasminogen colocalized with fibrin in WT mice, with a similar trend in KIT^W-sh^ mice (supplemental Figure 3B-D), suggesting a prothrombotic pulmonary environment during sepsis. Although oxygen saturation remained high in all experimental groups, likely due to oxygen-rich anesthesia, the histological findings reveal substantial pulmonary involvement that may have clinical relevance for sepsis-associated acute respiratory distress syndrome.

**Figure 3.**
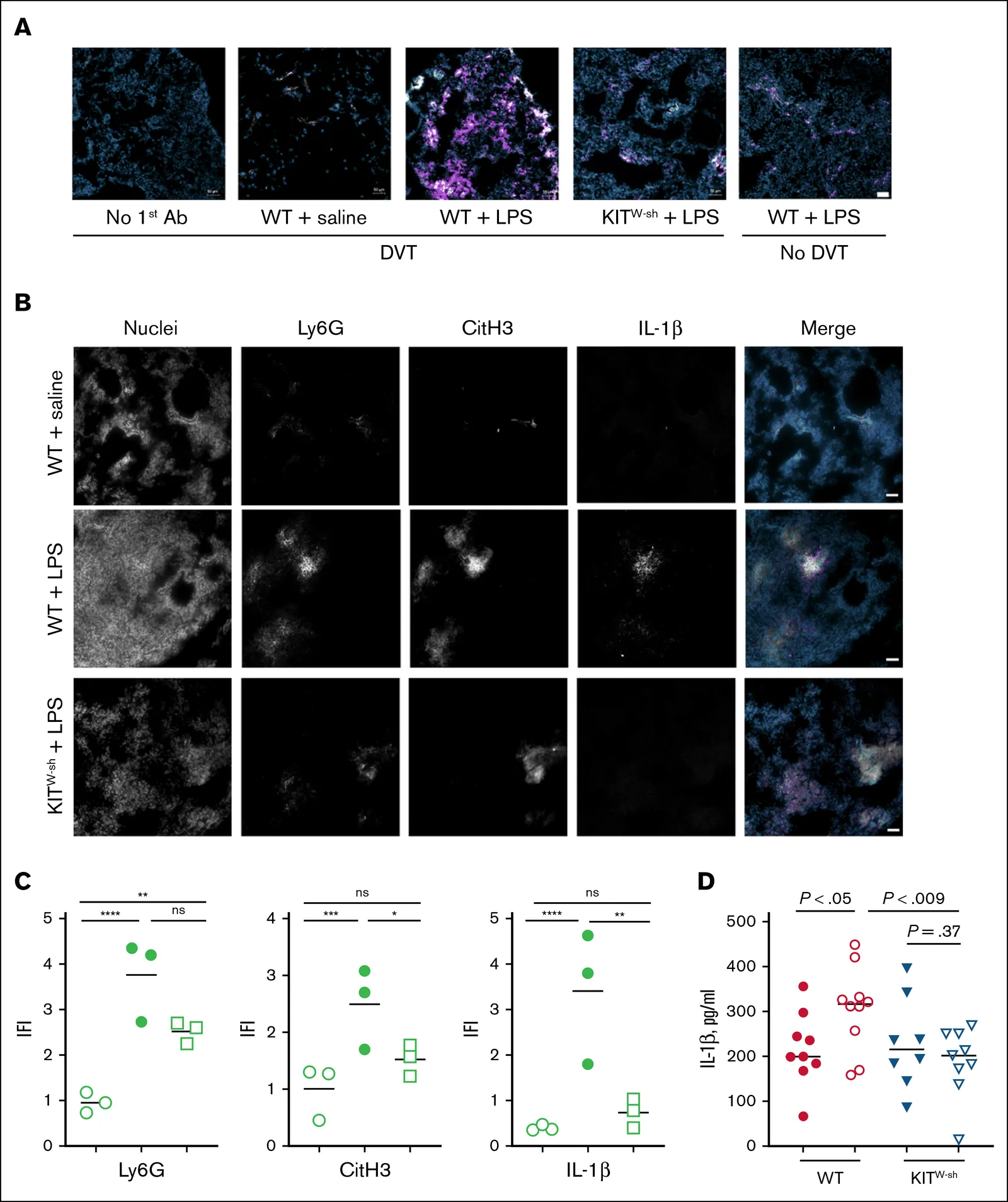
**MC deficiency reduces fibrin deposition, NETs, and IL-1β in lungs and IL-1β in plasma in septic DVT.** (A) Immunostaining of the lungs from WT and KIT^W-sh^ mice after administration of saline or LPS (4 mg/kg) followed by 6 hours DVT (4 left panels) or LPS without DVT (right panel). Green, CD41/platelets; magenta, fibrin; blue, nuclei. A no-primary-antibody control is shown at far left; bar, 50 μm. (B) Staining of the lungs from mice after LPS + DVT for neutrophils (Ly6G), CitH3, and IL-1β. In the merge panel: blue, nuclei; red, Ly6G; green, CitH3; magenta, IL-1β; bar, 50 μm. (C) Quantitation of IFI performed by ImageJ. Open circles, WT + saline; filled circles, WT + LPS; open squares, KIT^W-sh^ + LPS; horizontal bar represents mean; n = 3. (D) Plasma levels of IL-1β measured by enzyme-linked immunosorbent assay after 6 hours of IVC stenosis in saline-treated mice (filled shapes) and LPS-treated animals (open shapes); (C-D) 1-way ANOVA with Tukey multiple-comparisons test; ∗*P* < .05, ∗∗*P* < .02, ∗∗∗*P* < .01, ∗∗∗∗*P* < .004; n = 3 (A-C) and 8 to 10 (D). Ab, antibody; ns, nonsignificant.

### Plasma inflammatory profile in septic DVT

Interestingly, MC deficiency did not globally suppress the cytokine response to endotoxin. Plasma analyses revealed reduced levels of angiopoietin-2 (Ang-2) in MC-deficient mice during septic DVT, whereas most other cytokines were unchanged (supplemental Figure 4). Ang-2 is a well-established mediator of endothelial activation and vascular permeability in sepsis.^20^ Elevated Ang-2 disrupts Tie2 signaling, promotes leukocyte adhesion, and can directly enhance NET formation.^21^ However, because this study was not designed to investigate mechanisms regulating endothelial Ang-2 release, these findings should be interpreted as associative rather than mechanistic. Future studies will be required to determine whether MC-derived mediators directly regulate endothelial Ang-2 release or whether reduced Ang-2 levels in KIT^W-sh^ mice reflect secondary attenuation of systemic thromboinflammation.

## Discussion

Our results support a revised view of MC function in venous thrombosis. Rather than acting primarily as initiators of septic DVT, MCs appear to operate during the remodeling phase of thrombus development, where leukocyte recruitment, NET release, and fibrin cross-linking progressively increase resistance to fibrinolysis. By promoting NET accumulation within thrombi and modulating endothelial signaling pathways, MCs shape the inflammatory “signature” of the thrombus and its susceptibility to enzymatic degradation while also influencing distant organ responses. This distinction between thrombus quantity (initiation and size) and thrombus quality (composition and lytic resistance) may help explain why some patients with septic responds poorly to standard thrombolytic therapies despite comparable clot burden. The persistence of similar thrombus prevalence at 24 hours in WT and KIT^W-sh^ mice further supports the concept that MCs are not major determinants of thrombus quantity during septic DVT, whereas the markedly reduced clinical severity observed in MC-deficient animals highlights their contribution to systemic thromboinflammatory responses.

A limitation of this study is the reliance on KIT^W-sh^ mice, which are known to exhibit abnormalities beyond MC deficiency. Consequently, although our findings are consistent with previously established MC-dependent mechanisms in venous thrombosis and support a role for MCs in regulating thrombus composition and thromboinflammatory responses during endotoxemia, they do not by themselves establish definitive MC-specific causality. Future studies employing MC reconstitution or complementary MC-specific genetic models will therefore be important to confirm the altered NET accumulation and thrombolytic susceptibility observed in septic DVT. In addition, some mechanistic experiments were performed with limited sample sizes and should therefore be interpreted as exploratory observations requiring further validation in larger cohorts. Although the increased thrombolytic susceptibility of thrombi from KIT^W-sh^ mice suggests altered thrombus stability, the present study was not designed to evaluate long-term thrombus resolution in septic DVT. Future longitudinal studies will be required to determine whether MC deficiency accelerates spontaneous thrombus remodeling or clearance in septic DVT.

Overall, our findings identify MCs as key regulators of thrombus stability and systemic thromboinflammation during sepsis. Therapeutically, strategies targeting MC-driven NET formation or combining DNase with tPA may enhance thrombolysis, whereas modulation of the Ang-2/Tie2 pathway could mitigate pulmonary complications. Overall, these results shift the perspective from MCs as initiators of septic DVT toward MCs as determinants of thrombus behavior and inflammatory propagation, emphasizing their potential as targets for adjunctive therapies aimed at improving clot resolution and reducing organ injury.

Conflict-of-interest disclosure: The authors declare no competing financial interests.

## Supplementary Material

Supplemental Methods, References, and Figures
