# Supplementary material for: Mast cells enhance venous thrombus stability and lung fibrin deposition in murine sepsis
Source: Blood Adv. 2026 Sep 4;10(20):7166–73. doi: 10.1182/bloodadvances.2026020424 (PMC13692141; doi:10.1182/bloodadvances.2026020424)
Supplement: Supplementary file 1 [file BLOODA_ADV-2026-020424-mmc1.pdf]

METHODS.

Mice.

$KIT^{W\text{-}sh}$ mice were from Jackson lab on C57BL/6 background and were maintained as a homozygous breeding colony. Wild-type controls were age-, weight- and sex-matched C57BL/6 mice. All animals were housed under identical specific pathogen-free conditions and experiments were performed contemporaneously. All animal experiments were performed in accordance with the Animals Scientific Procedures Act 1986, conform to the guidelines from Directive 2010/63/EU of the European Parliament on the protection of animals used for scientific purposes, and were approved by the local Ethical Committee and the UK Home Office (project license PP8612246).

DVT surgery.

Stenosis of the IVC was applied as described.[1] Briefly, mice received intraperitoneal injection of LPS (Sigma, 4 mg/kg) 1 h prior to surgery. The dose and timing of LPS administration were selected to induce a reproducible acute systemic inflammatory (endotoxemic) response and thrombo-inflammatory activation while maintaining sufficient physiological stability to perform the technically demanding IVC stenosis surgery. This model was intended to mimic acute endotoxemia-associated thrombo-inflammation rather than the full complexity of human polymicrobial sepsis. Mice were given subcutaneously 0.1 mg/kg buprenorphine as an analgetic 1 h before surgery and then every 12 h. Anaesthesia was induced with 4% isoflurane in 100% oxygen (flow rate 1 L/min) and maintained at 2.0-2.5% isoflurane in 100% oxygen (flow rate 0.8 L/min) via a nose cone throughout surgery. Animals were placed in the supine position on a heated (30°C) surface. Middle abdominal incision was performed, guts were gently exteriorized and covered by gauze soaked in warm saline. IVC was gently separated from aorta at the site of abdominal IVC fusion with the left renal vein and closed with 7-0 polypropylene suture over a space (30G needle), after which the space was removed. This procedure results in ~90% closure of the IVC lumen. All side branches of the IVC were ligated. Then the incision was closed by a silk 6-0 suture and mice were allowed to wake up. In 6 h, mice were humanely culled by cervical dislocation, and thrombi and lungs were excised for analysis.[2]

Systemic clinical score to assess the severity of endotoxemia in LPS-mediated sepsis.

Clinical score was determined based on each animal's appearance, spontaneous activity, response to stimulation and eye opening as described.[2]

Immunostaining.

Frozen sections were fixed in acetone for 20 min, dried, washed with 0.05% Tween and incubated with ammonium chloride (20 mM, 15 min) to quench autofluorescence. Next, sections were washed, permeabilized with 0.5% Triton, and blocking was performed by 1% Bovine Serum Albumin (BSA, 1 h, room temperature). Thereafter, slides were incubated with anti-CD41 monoclonal antibody (MWReg30, rat anti-mouse, BD Pharmingen, BD Biosciences, San Diego, CA, USA), anti-Ly-6G/Ly-6C monoclonal antibody (RB6-8C5, rat anti-mouse, Invitrogen, Thermo Fisher Scientific, Waltham, MA, USA), goat anti-mouse anti-

fibrin/fibrinogen (no. YNGMFBG7, Accurate Chemical and Scientific Corporation, NY, USA; 1:10,000 in blocking solution), rabbit polyclonal anti-plasminogen antibody (Novus Biologicals, a Bio-Techne brand, NB300-544, Bio-Techne Ltd, UK), polyclonal goat anti-mouse S100A9 (R&D Systems, Bio-Techne, Minneapolis, MN, USA), polyclonal rabbit anti-mouse MPO (Thermo Fisher Scientific, Waltham, MA, USA), polyclonal goat anti-mouse IL-1β (R&D Systems, Bio-Techne, Minneapolis, MN, USA), or anti-histone H3 antibody (citrulline R2 + R8 + R17, rabbit anti-mouse, Abcam, ab281584, 1:100) overnight at 4°C. Then, after washing, secondary antibodies (anti-rabbit AF488, anti-rat AF568, anti-goat AF Plus 647, all 1:1,500 in PBS, for 45 min at room temperature) were applied. Staining for nuclei was performed by Hoechst 33342 trihydrochloride, trihydrate (Life tech H3570) 1:10,000 in PBS (5 min, room temperature). Slides were mounted using Prolong Gold antifade reagent (Invitrogen P36930), coverslips were sealed with nail varnish and allowed to dry for 2 hours before imaging. Slides were analyzed and images taken using epifluorescent microscope and a Zeiss Axio Scan Z1 Slide Scanner. Images for Supplementary Figure 2 were acquired by EVOS S1000 Imaging System followed by spectral unmixing for DAPI, Alexa 488, 594 and 647. Integrated Fluorescence Intensity (IFI) and area were evaluated using ImageJ (NIH, USA).[3]

Ex-vivo thrombolysis.

Thrombi were placed in the solution of tPA (3 μg/ml) or DNase I (40 U/ml, Stemcell Technologies, Cambridge, UK) or both in 1 ml saline and incubated on a shaker (400 oscillation/min, 3 h, 30°C). Thrombi were briefly placed on absorbent paper to remove excess surface liquid, after which their wet weight was measured. This procedure was repeated at each 30-minute time point throughout the thrombolysis assay. Results are expressed as the percentage of the initial thrombus weight. Results are presented as percentage of the initial thrombus weight.

Pulse oximetry.

MouseOx Small Animal Vital Signs Monitor (pulse oximeter, Starr Life Sciences Corp. PA, USA) was used for pulse oximetry. Anaesthesia was induced with 4% isoflurane in 100% oxygen (flow rate 1 L/min) and maintained at 2.0–2.5% isoflurane in 100% oxygen (flow rate 0.8 L/min) via a nose cone the procedure. The procedure was performed by placing a small, non-invasive probe on the neck of the anesthetized mouse. The probe was positioned securely to ensure stable readings, minimizing motion artifacts. Measurements were recorded during 10 s while the mouse was anesthetized with an isoflurane-oxygen mixture prior to surgery and before culling the animal in 6 h to assess oxygenation levels during the experiment.

Measurement of inflammatory plasma markers

Plasma levels of IL-1β were measured by ELISA. PCSK9, angiopoietin-2, CXCL-12, CCL5, IL-10, S100A9, PAI-1, TNFα, CCL2, CXCL-1, ICAM-1, IL6 and CD62P were measuring using luminex (R&D).

Statistics

Data are presented as individual data points or mean ± SD, as indicated in the corresponding figure legends. Graphs were created using GraphPad Prism 9 (GraphPad Software Inc., La Jolla, CA, USA). Statistical analyses were performed in GraphPad Prism 9 using contingency tables followed by Fisher's exact test (for binary data, such as thrombosis prevalence), Student's t-test (for comparing single values), or one-way ANOVA with corrections for multiple comparisons, and differences were considered statistically significant at $p < 0.05$. Because several exploratory experiments involved small sample sizes ($n = 3$), key findings were additionally confirmed using non-parametric Kruskal-Wallis test with Dunn's multiple-comparison correction analysis, which yielded similar conclusions.

References

1. Payne H, Brill A. Stenosis of the Inferior Vena Cava: A Murine Model of Deep Vein Thrombosis. *J Vis Exp*. 2017(130).
2. Rayes J, Lax S, Wichaiyo S, et al. The podoplanin-CLEC-2 axis inhibits inflammation in sepsis. *Nat Commun*. 2017;8(1):2239.
3. Schneider CA, Rasband WS, Eliceiri KW. NIH Image to ImageJ: 25 years of image analysis. *Nat Methods*. 2012;9(7):671-675.

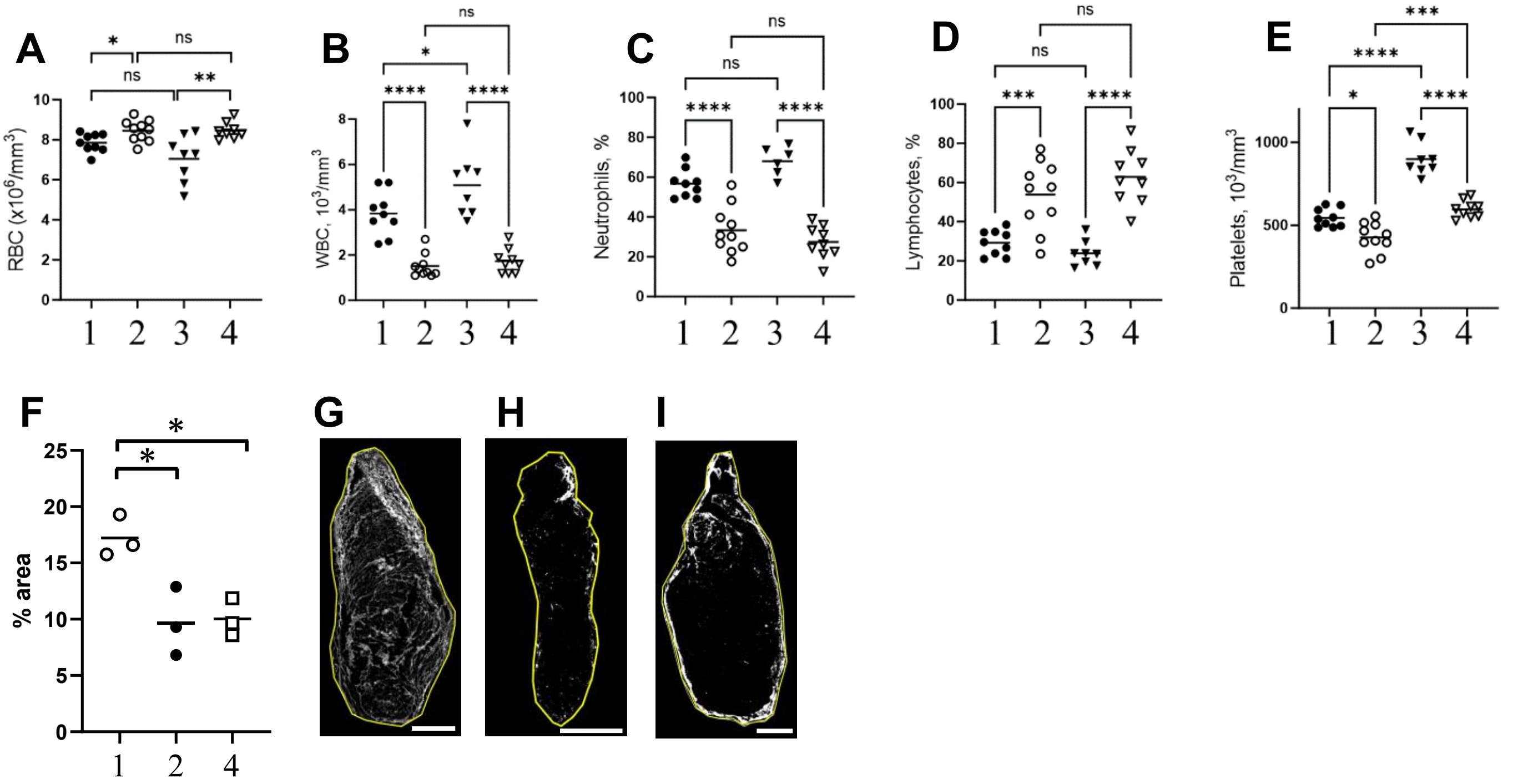

Supplementary Fig 1. Blood counts and intra-thrombus neutrophils of mice after LPS-DVT. (A - E) Blood was drawn 6 h after DVT induction following injection of either saline or LPS (4 mg/kg). Groups are: 1, WT + saline; 2, WT + LPS; 3, $KIT^{W\text{-}sh}$ + saline; and 4, $KIT^{W\text{-}sh}$ + LPS. (F) Thrombus sections were stained for Ly6G, a neutrophil marker, and the Ly6G-positive area was quantified as a percentage of the total thrombus area. Representative images of Ly6G staining corresponding to the groups quantified in (F): (G) WT + saline (group 1); (H) WT + LPS (group 2); and (I) $KIT^{W\text{-}sh}$ + LPS (group 4); Yellow outlines indicate the thrombus area used for quantification. Statistical analysis was performed using one-way ANOVA with Tukey's multiple-comparisons test, ns, non-significant. * $p < 0.05$, ** $p < 0.02$, *** $p < 0.01$, **** $p < 0.004$. Scale bar, 1 mm; n = 8 - 10 per group (A - E) and n = 3 per group (F - I).

RBC, Red Blood Cells; WBC, White Blood Cells

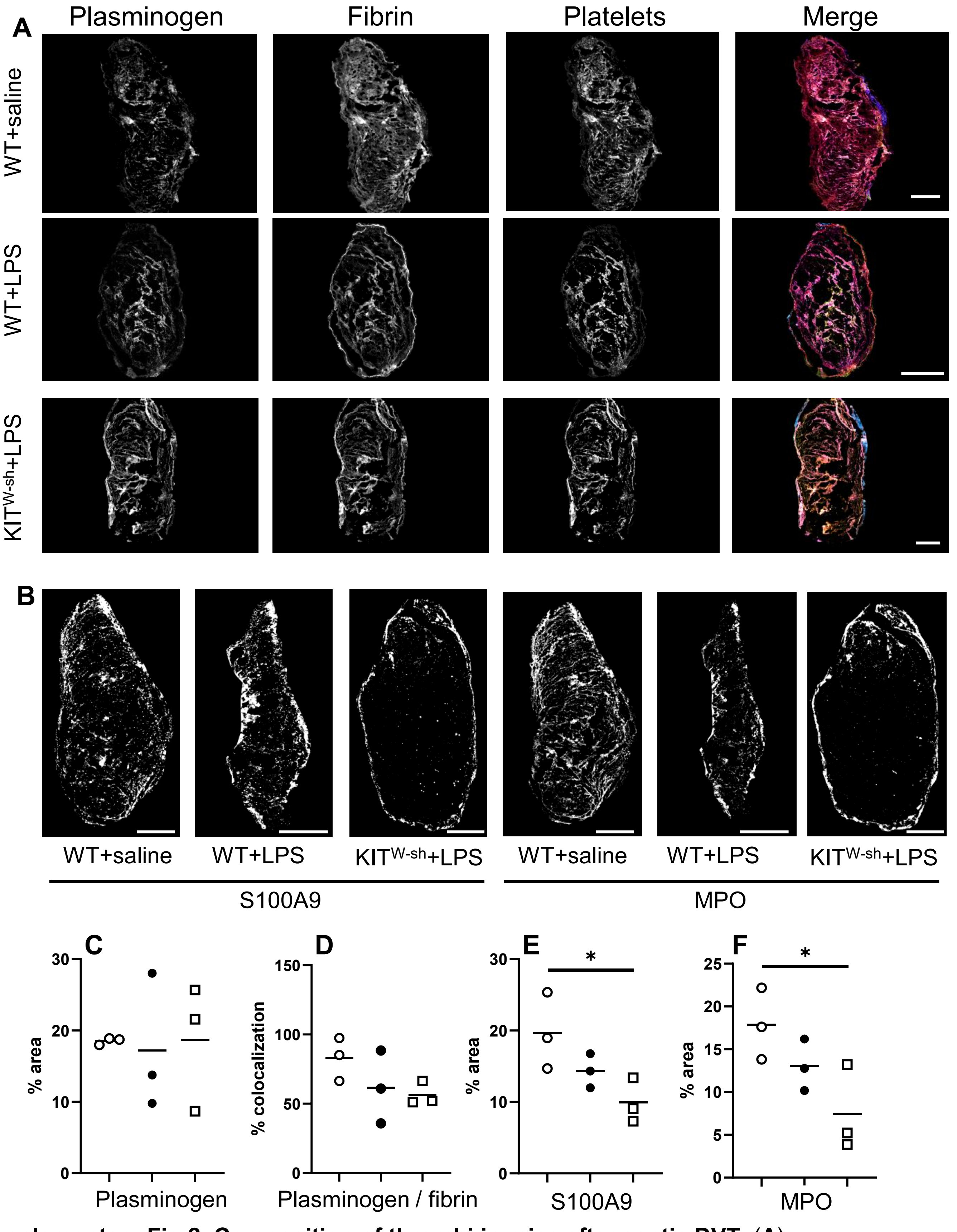

Supplementary Fig 2. Composition of thrombi in mice after septic DVT. (**A**) Representative staining for plasminogen, fibrin and platelets (CD41) of thrombi from WT mice + saline or LPS and KIT^{W-sh} mice + LPS. Merged images: green, plasminogen; red, fibrin;, magenta, CD41, blue nuclei. (**B**), representative staining for S100A9 and MPO; scale bar 1 mm. (**C – F**) quantitation of staining for: C, plasminogen; D, % of colocalization of plasminogen with fibrin; E, S100A9; F, MPO. Open circles, WT + saline; black circles, WT + LPS; open squares, KIT^{W-sh} mice + LPS. Statistical analysis was performed using one-way ANOVA with Tukey's multiple-comparisons test; * $p < 0.05$; n = 3.

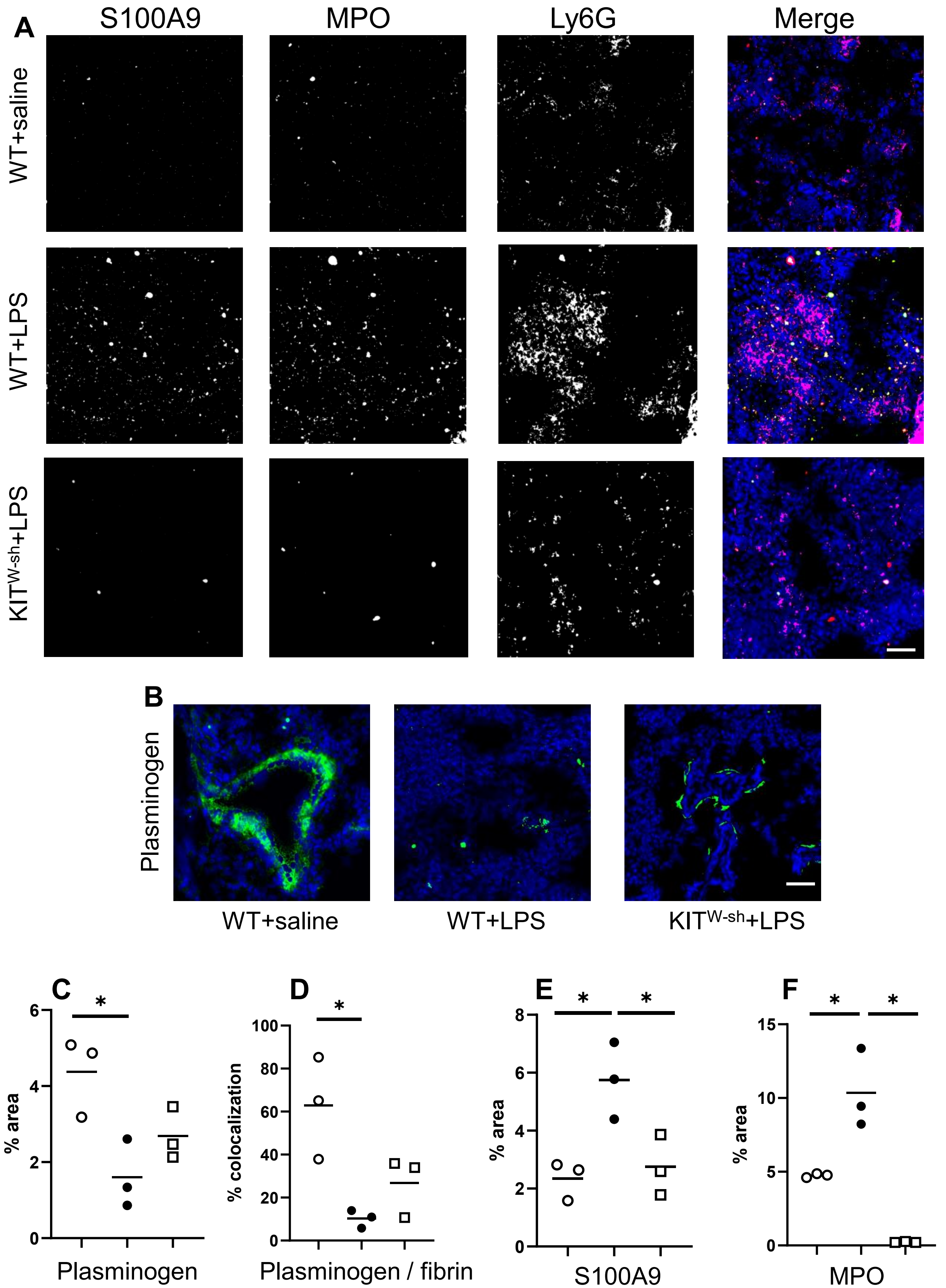

Supplementary Fig 3. Thromboinflammation in the lungs in mice after septic DVT. (**A**) Representative staining for S100A9, MPO and neutrophils (Ly6G) of lungs from WT mice + saline or LPS and KIT^{W-sh} mice + LPS. Merged images: green, S100A9; red, MPO; magenta, Ly6G, blue nuclei. (**B**), representative staining for plasminogen (green) and nuclei (blue); scale bar 50 μm. (**C – F**) quantitation of staining for: C, plasminogen; D, % of colocalization of plasminogen with fibrin; E, S100A9; F, MPO. Open circles, WT + saline; black circles, WT + LPS; open squares, KIT^{W-sh} mice + LPS. Statistical analysis was performed using one-way ANOVA with Tukey's multiple-comparisons test; * $p < 0.05$; n = 3.

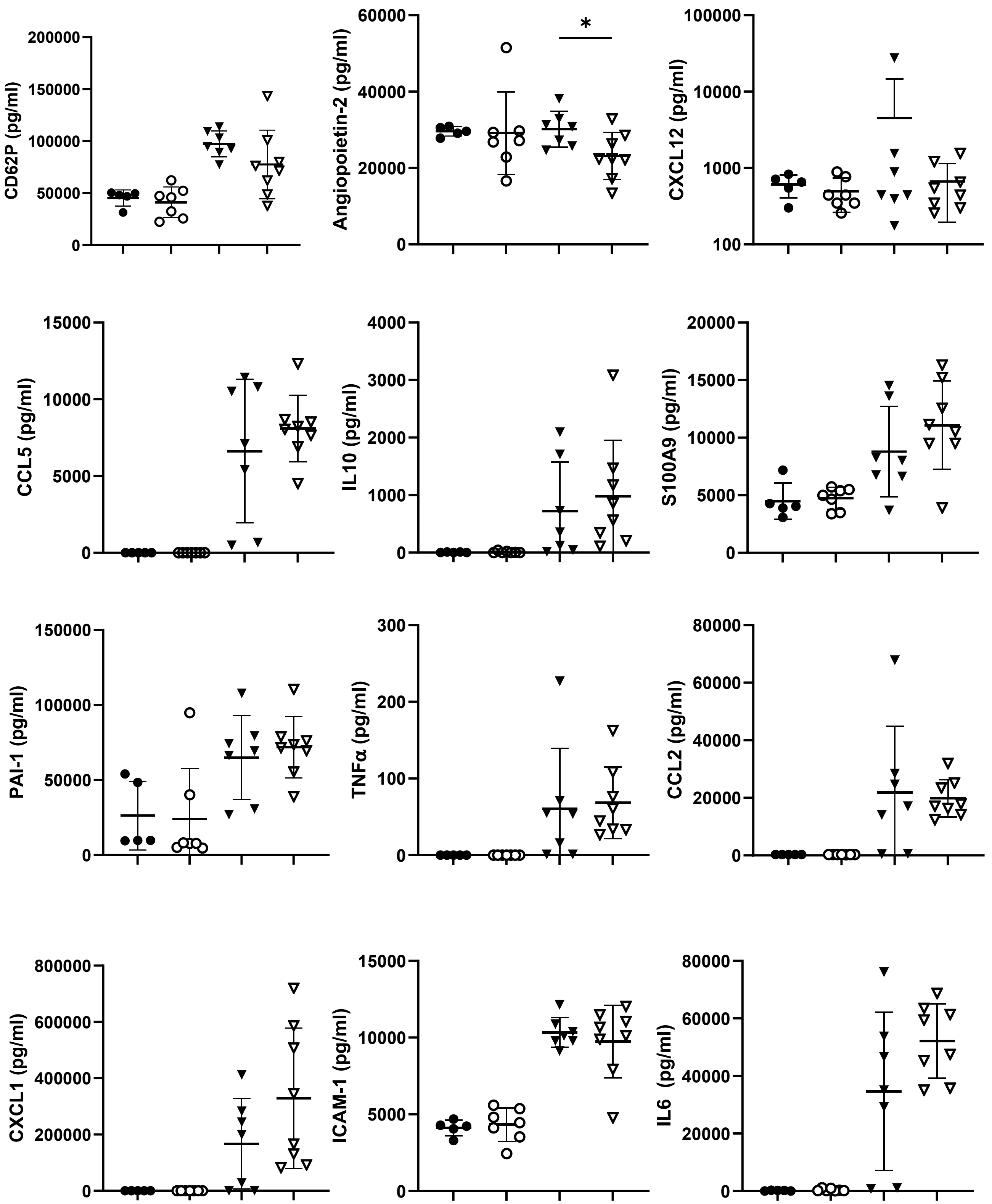

Supplementary Fig 4. Plasma inflammatory profile in mice after septic DVT. Wild-type or $KIT^{W\text{-}sh}$ mice were administered with either sterile saline or LPS (4 mg/kg) and then subjected to IVC stenosis for 6 h. Blood was drawn prior to culling the mice, and plasma levels of a variety of inflammation-related molecules were determined by Luminex. Black circles, WT+saline; open circles, $KIT^{W\text{-}sh}$+saline; black triangles, WT+LPS, open triangles, $KIT^{W\text{-}sh}$+LPS; Each symbol represents one mouse; horizontal bars indicate mean ± SD. Statistical analysis was performed using one-way ANOVA with Tukey's multiple-comparisons test, * $p < 0.05$; n = 5-8 mice per group.
